# Gone in 2 seconds: Ablation of a recurrent accessory pathway using pulsed field ablation

**DOI:** 10.1016/j.hrcr.2025.03.017

**Published:** 2025-03-27

**Authors:** Ven Gee Lim, Sandeep Panikker, Faizel Osman

**Affiliations:** 1 Department of Cardiology, University Hospitals Coventry and Warwickshire, NHS Trust, United Kingdom; 2 Institute for Cardio-Metabolic Medicine, University Hospitals Coventry and Warwickshire, United Kingdom; 3 Warwick Medical School, University of Warwick, United Kingdom

**Keywords:** Pulsed field ablation, Accessory pathway, Supraventricular tachycardia, Atrioventricular re-entry tachycardia, Electroanatomic mapping

**Key Teaching Points**

• Pulsed field ablation (PFA) has been widely used in the treatment of atrial fibrillation, but its use in the treatment of accessory pathway is limited.
• Case series have been described on the use of a focal PFA catheter in the ablation of accessory pathways. Our case is the first description of the feasibility, safety, and efficacy of a circular PFA catheter in the ablation of a recurrent right-sided accessory pathway.
• Longer follow-up for recurrences in this group of patients is warranted, as it will help shed some light on the long-term durability of lesions from PFA.

## Introduction

This case report describes the novel use of pulsed field ablation to treat an incessant supraventricular tachycardia secondary to a recurrent concealed accessory pathway.

## Case report

**Figure 1:**
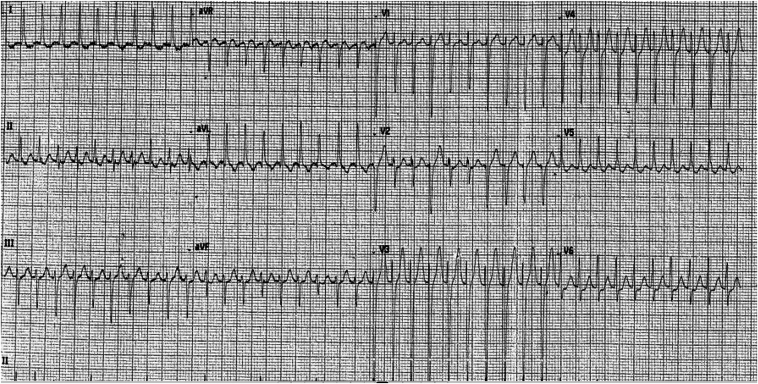
The initial electrocardiogram that documented a regular narrow complex tachycardia with a ventricular rate of about 240 bpm.

A 28-year-old man with a 1-year history of palpitations secondary to a supraventricular tachycardia (SVT) underwent an elective cardiac electrophysiologic (EP) study. He had a background of type 1 diabetes mellitus and was taking insulin and bisoprolol. His initial documented SVT demonstrated a regular narrow complex tachycardia with a ventricular rate of about 240 bpm (Figure 1).

**Figure 2:**
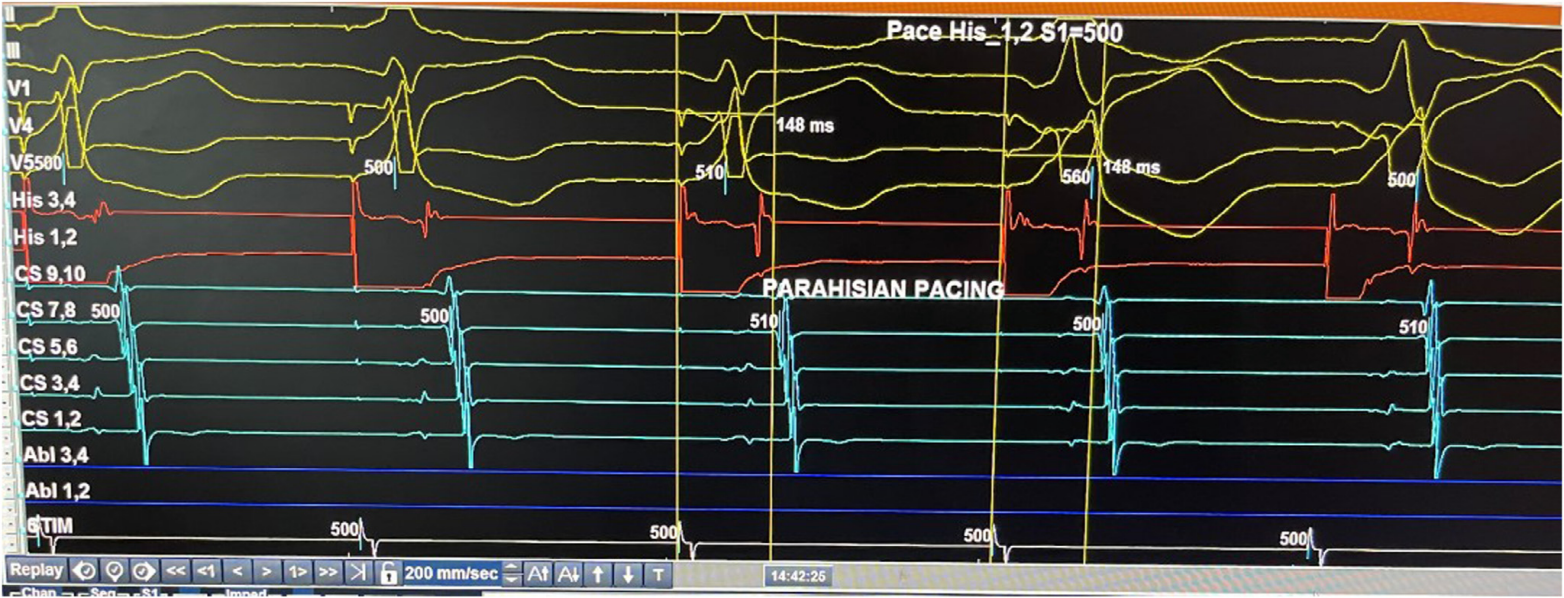
A Parahisian pacing maneuver showed an unchanged stimulus-atrial interval time of 148 ms when the pacing stimulus (from the red channel His 1, 2) capture was changed from the His bundle to the ventricular myocardium. This retrograde conduction response suggested the presence of an accessory pathway.

**Figure 3:**
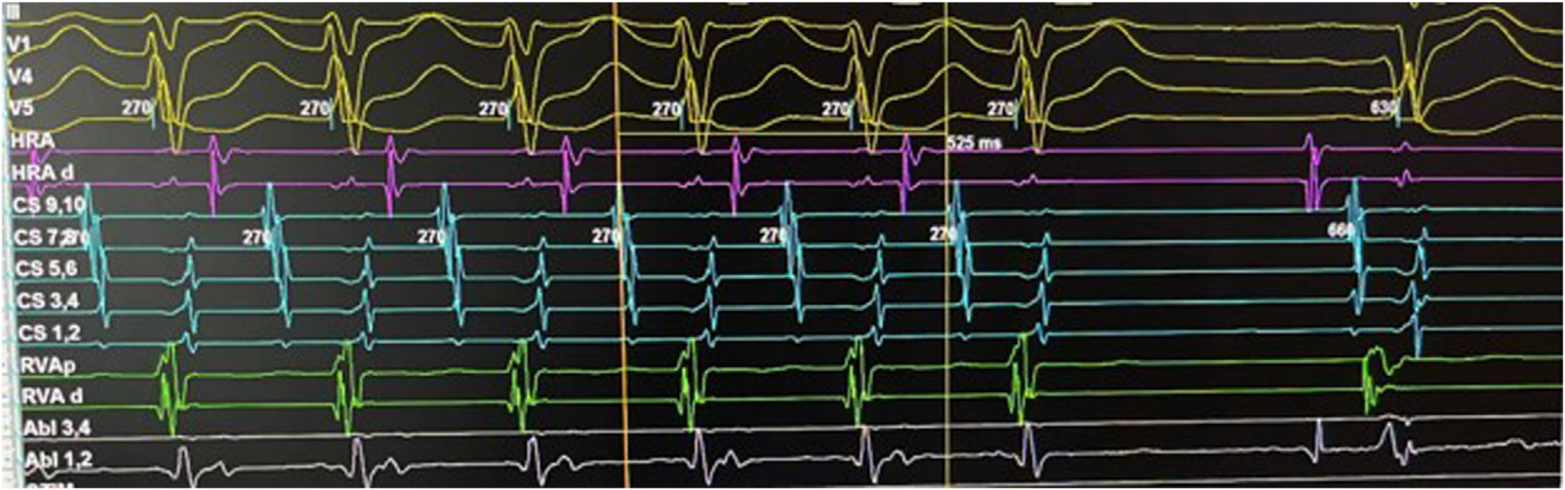
Termination of the narrow complex tachycardia (1:1 ventriculoatrial conduction with a long ventriculoatrial time) with radiofrequency ablation.

His initial 3-wire EP study (catheters marking the His, coronary sinus, and right ventricular apex) demonstrated concentric and non-decremental conduction on the retrograde curve. Parahisian pacing maneuvers demonstrated a pathway response (Figure 2). A fourth EP catheter was added to mark the high right atrium. During catheter manipulation, an incessant orthodromic atrioventricular (AV) reentry tachycardia secondary to a concealed right free wall accessory pathway was induced. The accessory pathway was successfully ablated during tachycardia with focal radiofrequency ablation (irrigated Flexibility ablation catheter; Abbott, Minneapolis, MN) and the intracardiac signals of tachycardia termination are shown in Figure 3. There was no evidence of recurrence after a 30-minute wait with demonstration of AV and ventriculoatrial block upon administration of adenosine. He was discharged the same day, and his bisoprolol was discontinued.

**Figure 4:**
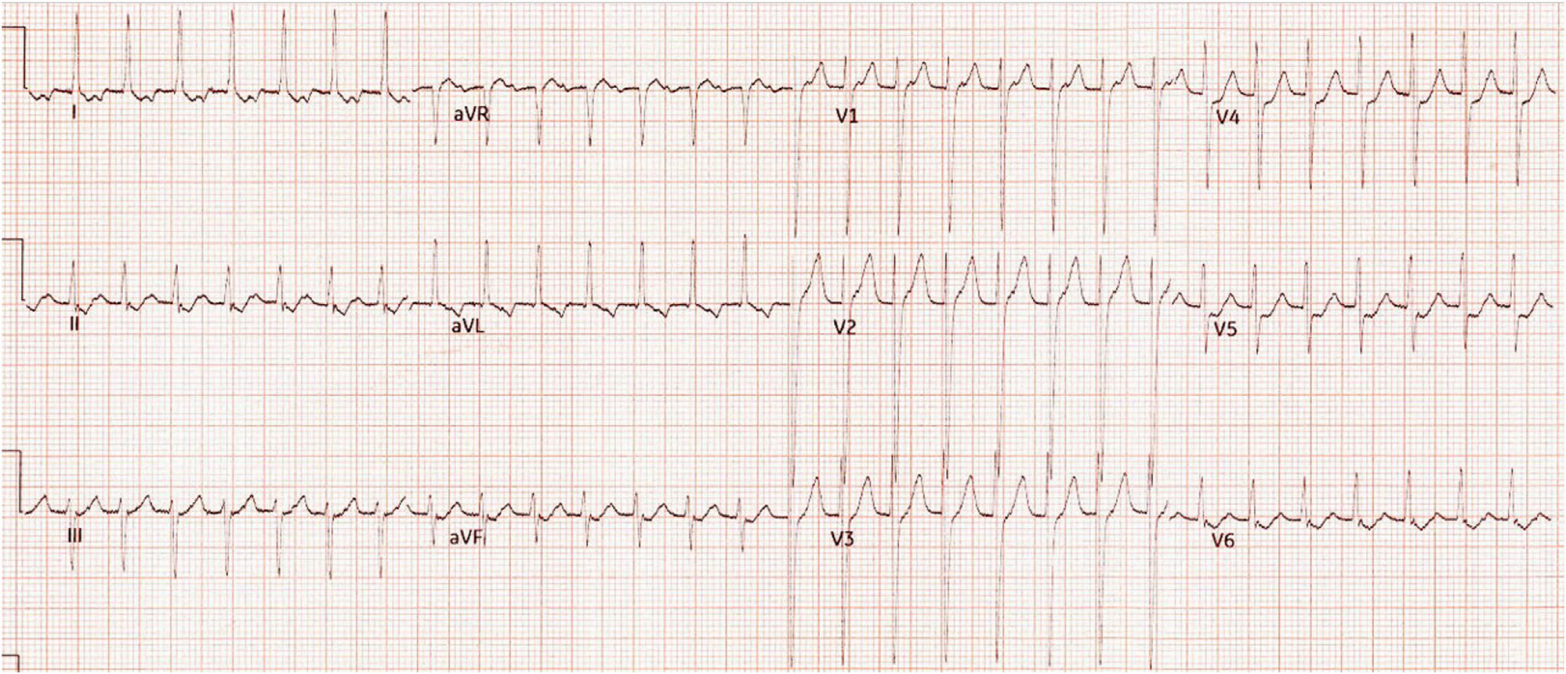
The electrocardiogram 6 months after accessory pathway ablation that documented a regular narrow complex tachycardia with a ventricular rate of about 180 bpm.

He experienced mild self-terminating palpitation episodes 1 week after ablation. At 6 months post-ablation, he was hospitalized for pulmonary oedema secondary to a sustained SVT (a ventricular rate of 180 bpm; Figure 4), which required an emergency electrical cardioversion. He was admitted to the intensive treatment unit for continuous positive airway pressure and intravenous diuresis.

**Figure 5:**
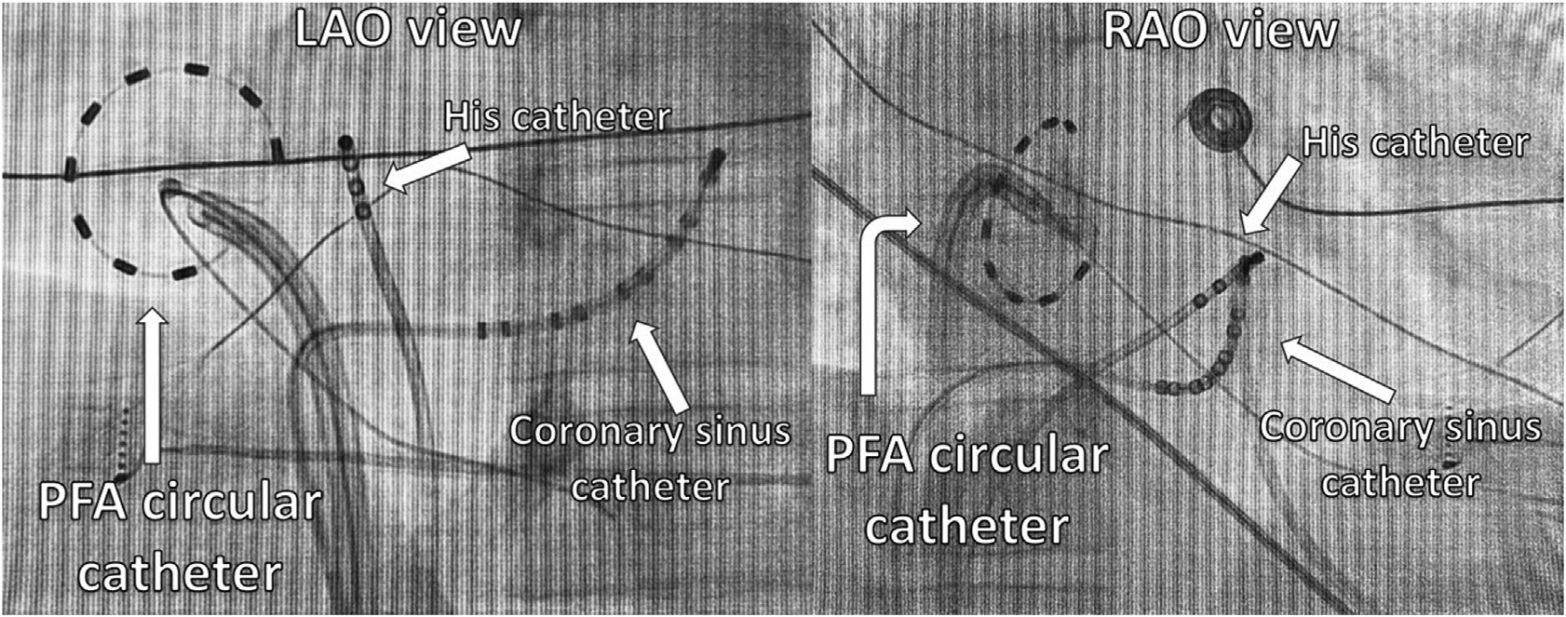
Positions of the pulsed field ablation (PFA) circular catheter, coronary sinus catheter, and His catheter on the left anterior oblique (LAO), and right anterior oblique (RAO) fluoroscopic views. Venous access was obtained via the right femoral vein, and the PFA circular catheter was delivered via a long support sheath.

**Figure 6:**
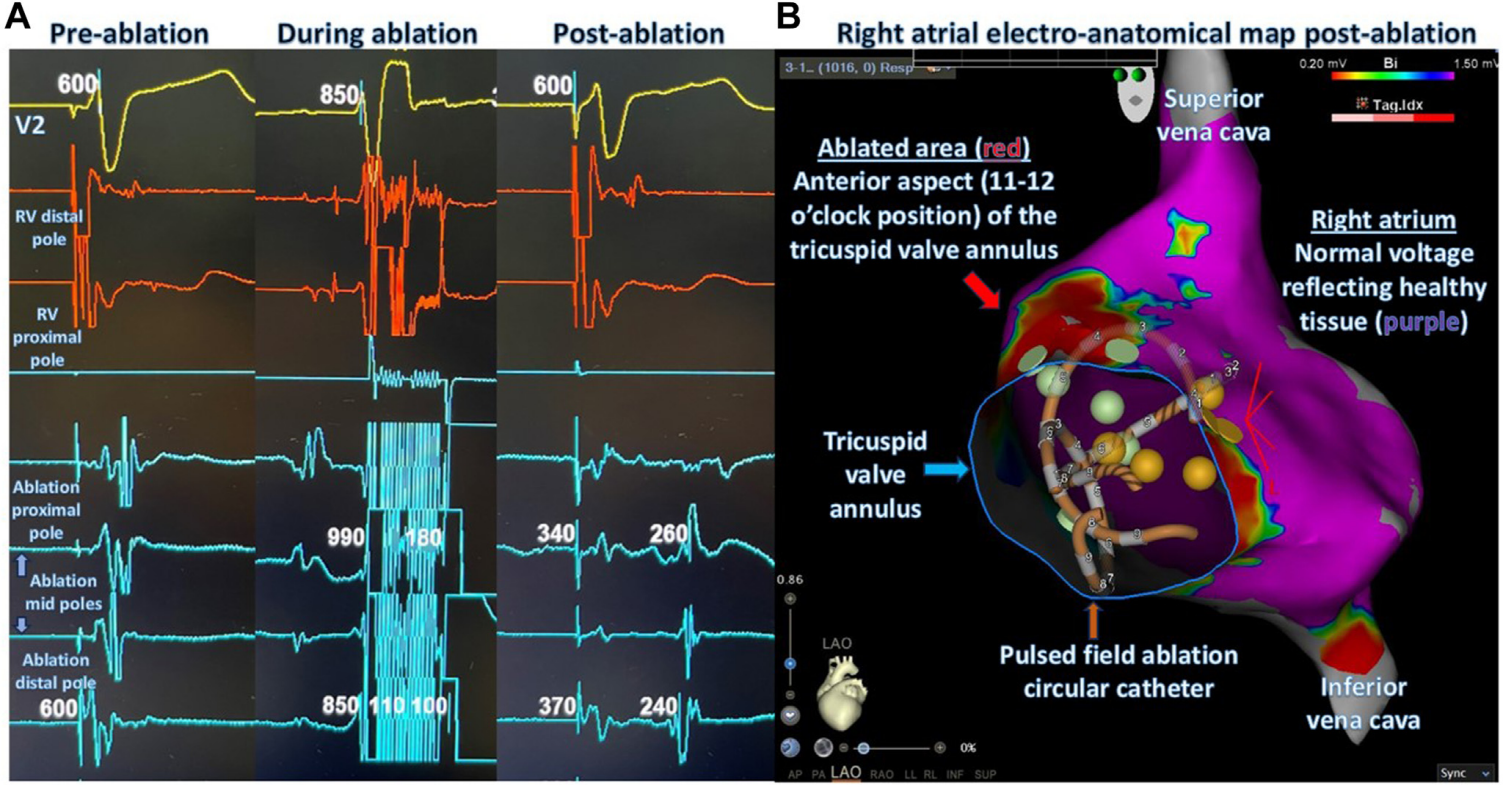
Accessory pathway ablation within 2 seconds of pulsed field ablation (PFA). **A:** Intracardiac electrogram that demonstrates loss of retrograde activation of the right atrium following the application of PFA. **B:** Three-dimensional electroanatomic map of the right atrium after ablation in the left anterior oblique view. The PFA catheter position is depicted (*orange arrowhead*). The *purple area* depicts normal voltage/healthy myocardial tissue, and the *red area* depicts the ablated region (*red arrowhead*).

Once the patient’s condition stabilized, he was moved to the cardiology ward, where he underwent an inpatient EP study to evaluate for a recurrence of his accessory pathway. Like the index EP study, this elicited a concentric non-decremental response on the retrograde curve and a pathway response on para-Hisian pacing. With 3-dimensional electroanatomic mapping (Carto 3, Biosense Webster, Irvine, CA), a concealed right free wall accessory pathway localized at the 11- to 12-o’clock position on the tricuspid valve annulus was demonstrated. The His position was tagged on fluoroscopy (Figure 5) and 3-dimensional mapping. Pulsed field ablation (PFA) using a 9-electrode circular catheter (PulseSelect, Medtronic, Minneapolis, MN) via a long sheath (FlexCath Contour, Medtronic) was applied to the earliest breakout location on the tricuspid valve annulus. Within 3 PFA applications (1.8 seconds in total), termination of retrograde conduction via the accessory pathway was achieved (Figure 6). There was no evidence of recurrence after a 30-minute wait with demonstration of AV and ventriculoatrial block upon administration of adenosine and a nodal response on para-Hisian pacing. Coronary angiography was not performed, but there were no ischemic changes on the 12-lead ECG before, during, and after PFA application. The total fluoroscopy time was 9 minutes 33 seconds and dose area product was 2.28 Gy·cm^2^.

The patient was discharged the following day with discontinuation of oral bisoprolol and flecainide. He has not experienced any symptom recurrence at 3-months follow-up.

## Conclusion

Two groups have reported the use of a focal PFA catheter in the ablation of accessory pathways.^1^^,^^2^ However, we believe that our case is the first description of the feasibility, safety, and efficacy of a circular PFA catheter in the ablation of a recurrent right-sided accessory pathway. Longer follow-up for recurrences in this group of patients will help to shed some light on the long-term durability of lesions from PFA. Further study is warranted to evaluate the utility of different PFA catheters in the ablation of accessory pathways.

## Disclosures

The authors have no conflicts to disclose.
